# Timing of Surgery for Asymptomatic Primary Mitral Regurgitation: Possible Value of Early, Serial Measurements of Left Ventricular Sphericity

**DOI:** 10.2174/011573403X277223240206062319

**Published:** 2024-02-13

**Authors:** Alfred Stanley, Constantine Athanasuleas

**Affiliations:** 1 Cardiovascular Associates of the Southeast, Birmingham AL and Kemp-Carraway Heart Institute, Birmingham, AL, USA;; 2 Department of Surgery, North Alabama Medical Center and Kemp-Carraway Heart Institute, Birmingham, AL, USA

**Keywords:** primary mitral regurgitation, left ventricular sphericity, timing of mitral valve repair, left ventricular shape, myxomatous degeneration, diastolic filling

## Abstract

Asymptomatic primary mitral regurgitation due to myxomatous degeneration of the mitral valve leaflets may remain so for long periods, even as left ventricular function progresses to a decompensated stage. During the early compensated stage, the ventricle’s initial response to the volume overload is an asymmetric increase in the diastolic short axis dimension, accomplished by a diastolic shift of the interventricular septum into the right ventricular cavity, creating a more spherical left ventricular diastolic shape, increasing diastolic filling and stroke volume. Early valve repair is recommended to reduce postoperative left ventricular dysfunction. Early serial measurements of left ventricular sphericity index [LV-Si]. during the compensated stage of mitral regurgitation might identify subtle changes in left ventricular shape and assist in determining the optimal earliest timing for surgical intervention.

## 1 INTRODUCTION

Mitral regurgitation [MR], is defined as systolic retrograde blood flow from the left ventricle through the mitral valve into the left atrium [1]. The most common cause of primary MR is myxomatous degeneration of the mitral valve leaflets [2]. The established contemporary therapeutic strategy for patients with severe primary MR is early surgical mitral valve repair [3, 4].

This manuscript reviews asymptomatic primary MR and its effect on left ventricular (LV), remodeling with an emphasis on ventricular shape. The left ventricular sphericity index (LV-Si), is a measurement of LV shape and is quantified by three-dimensional (3D). echocardiography. The purpose of this review is to examine the first indication of ventricular remodeling as the left ventricle adapts to the volume overload from primary mitral regurgitation. We report the earliest left ventricular remodeling known to be associated with primary mitral regurgitation [5, 6]. We review left ventricular shape changes during the compensated stage of mitral regurgitation and speculate that early serial measurements of the LV sphericity index may identify the left ventricles' earliest reversible remodeling response before the development of irreversible ventricular dysfunction. Identifying this time point in the progression of mitral regurgitation would provide the optimal timing for surgical intervention for asymptomatic patients with severe primary mitral regurgitation.

## 2 VENTRICULAR CONSEQUENCES OF PRIMARY MITRAL REGURGITATION

The human heart has an exceptional ability to alter its phenotype to adapt to changes in environmental demand. This allows the heart to operate across a wide range of contractile demands for the short term and chronically adjust the cardiac structure and function in response to constant exposure to excessive pressure and/or volume overload. A persistent change in the heart’s phenotype is termed cardiac remodeling or plasticity. This complex and multifactorial process transforms the LV shape, size, and wall thickness, all driven by mechanical load and the neurohormonal axis [7-9]. As MR progresses, the heart changes its shape and becomes more spherical [10].

The heart adapts to an increased, sustained pressure/volume load by augmenting muscle mass. The mass increase is due to hypertrophy of existing myocytes rather than hyperplasia because cardiomyocytes become terminally differentiated soon after birth [11, 12]. Hypertrophy is a stress-specific response [13]. The pattern of remodeling is determined by the type of overload [14, 15]. Grossman described the left ventricle’s remodeling response to various hemodynamic overloads in 1975 [15]. Pressure and volume overload result in divergent patterns of hypertrophy. In response to increased afterload [pressure overload], there is a parallel addition of sarcomeres that increases myocyte width, which increases wall thickness. This type of remodeling results in concentric hypertrophy and an increased wall thickness-to-cavity radius ratio (h/R). According to La Place’s law, increased pressure-overload wall stress is counterbalanced by increase wall thickness. In contrast, the response to increase preload (volume overload) is myocyte lengthening by sarcomere replication in series and an increase in ventricular volume. This pattern of remodeling causes progressive eccentric hypertrophy, cavity dilation and inadequate wall thickening to reduce wall stress, resulting in a normal wall thickness-to-cavity radius ratio (h/R). This remodeling allows the heart to sustain a high stroke volume but does not counterbalance the increased diastolic wall stress [16, 17].

During mitral valve regurgitation, an excessive preload is imposed on the LV. Throughout systole, a regurgitant volume of blood is displaced back into the lower-pressure left atrial chamber. Ventricular filling of the next heart contraction is increased, resulting in volume overload. As an early compensatory response, eccentric hypertrophy develops to preserve forward stroke volume in the face of excess volume and increased chamber size [9, 18, 19]. This pattern of remodeling is thought to be driven by increased diastolic wall stress [15]. According to La Place’s law, chamber enlargement leads to an increase in wall stress caused by the mismatch in wall thickness-to-chamber radius ratio. Myocyte lengthening improves the efficiency of the ventricular chamber but fails to normalize diastolic wall stress [15, 16]. Several authors have reported a decrease in mass-to-volume ratio with volume-overloadand uncompensated wall stress leads to maladaptive remodeling that progresses insidiously [19-21].

Grossman’s 1975 assessment of cardiomyocyte remodeling as it relates to LV remodeling was later confirmed by histomorphometric measurements. In concentric hypertrophy, myocytes only grow in a transverse direction while keeping cell length constant, whereas in eccentric hypertrophy, cardiomyocytes grow proportionately in both longitudinal and transverse directions [22].

## 3 TIMING OF SURGERY

The timing of surgery in asymptomatic severe primary MR is a complex issue that has been extensively studied and debated [23]. Severe asymptomatic primary MR enlarges the left atrium (LA). There is a good correlation between left atrial emptying volume and mitral regurgitant fraction (r=0.85, *p*<0.01). [24]. The increase in left atrial wall compliance, resulting from atrial chamber enlargement, is enough to normalize the left atrial pressure and reduce or prevent clinical symptoms, even with severe MR [25]. This may explain why many patients with severe primary MR are asymptomatic at diagnosis yet are at risk for long-term LV dysfunction when treated medically [26]. The asymptomatic clinical picture is deceiving and originally led some to believe severe primary MR was benign and could be managed with a “watchful waiting” strategy, where patients were referred to surgery only when symptomatic or with LV enlargement or dysfunction [27, 28]. Patients with preoperative symptoms or overt LV dysfunction have excess short and long-term postoperative mortality following surgery [29]. Once LV dysfunction occurs, MR has already transitioned into a decompensated stage, and the prognosis is worse [30].

Natural history studies in patients with initially asymptomatic primary MR, treated with a “watchful waiting” surgical strategy, suggested that the long-term clinical course of these patients might be more problematic than previously thought [31, 32]. Numerous long-term surgical outcome studies reported poor late results when a ‘wait and see’ strategy was used to guide surgical timing [29, 33-35]. These data suggest early surgery for asymptomatic severe primary MR should be considered before ventricular enlargement develops, despite the clinical status. Indeed, many asymptomatic patients already have limited cardiopulmonary performance and impaired cardiac reserve which predicts the development of late postoperative LV dysfunction [36, 37]. There are also early asymptomatic alterations in left ventricular shape, increased diastolic sphericity and decreased conicity in patients with severe primary mitral regurgitation and LVEF > 55% [38]. MV repair significantly improves postoperative outcomes in patients with primary MR compared to replacement [3, 4, 39]. Mitral valve repair is associated with lower operative mortality, better long-term survival, and fewer valve-related complications [40].

Left ventricular global longitudinal strain [LV-GLS]. imaging *via* speckle tracking echocardiography has been advocated as an imaging technique to optimize the timing of early surgery [41, 42]. 2-D and 3-D speckle tracking echocardiography can be used to assess myocardial strain and strain rate, and measurements of myocardial deformation that reflect true ventricular muscle performance. However, subclinical LV dysfunction has been detected with LV-GLS imaging in patients with asymptomatic severe MR [41, 42]. An abnormal pre-operative LV-GLS has been shown to be a predictor of post-operative LV dysfunction [43, 44]. In addition, several recent meta-analyses suggest that in asymptomatic patients with severe MR and preserved LVEF, an impaired LV-GLS is a predictor of both LV dysfunction and long-term survival after mitral valve surgery [45, 46]. In many cardiac diseases, the first LV loss of myocardial function is in the longitudinal fibers of the endo and epicardial layers, while the circumstantial fibers in the mid-wall remain relatively unaffected or even compensate by augmenting circumferentially shortening that preserve the LVEF [45, 47-49]. Therefore, abnormal LV-GLS imaging indicates some degree of irreversible LV dysfunction has already occurred. A normal LV-GLS would confirm viability but would not identify early subclinical, reversible ventricular remodeling.

Guidelines for echocardiographic evaluation of mitral regurgitation are reported in the 2020 ACC/AHA Guidelines for the Management of Patients with Valvular Heart Disease: A Report of the American College of Cardiology/American Heart Association Joint Committee on Clinical Practice [50]. Recommendations for the timing of surgical intervention in asymptomatic patients with chronic primary MR are as follows: If regurgitation is severe and LVEF ≤ 60%, LVESD ≥ 40 mm, mitral surgery is recommended (Class I, LOE, B). MV repair is reasonable when the likelihood of successful repair is >95% with expected mortality < 1% (Class 2a LOE, B). Note, in reference to remodeling, although LV size and function are addressed, there is no mention of left ventricular sphericity. In the sections below, it is postulated that this measurement may be useful in identifying early, physiological, reversible remodeling before maladaptive LV enlargement and dysfunction develop.

## 4 QUANTIFYING MITRAL REGURGITATION

When considering early mitral valve surgery on asymptomatic patients, it is imperative to accurately quantify the severity of MR [51]. The 2020 ACC/AHA Guidelines for the Management of Valvular Heart Disease, mentioned above, also address the echocardiographic diagnostic and functional evaluation of primary mitral regurgitation [50]. Recommended testing to grade MR includes the semi-quantitative measurements of color flow, Doppler regurgitant jet area, the proximal isovelocity surface area (PISA), and the vena contracta (VC) All may encounter methodological problems and often fail to describe the associated hemodynamic conditions and Doppler quantification of MR by effective regurgitant orifice area (ERO) No single Doppler or echocardiographic measurement is adequate to grade MR. A comprehensive imaging examination should include two-dimensional (2D) and three-dimensional [3D]. transthoracic echocardiograms for structural evaluation and pulsed, color, and continuous wave Doppler for flow interrogation [52].

Echocardiography is operator-dependent, technically challenging and may not always give reliable views for differentiating severe from non-severe MR [53-56]. One study evaluated the interobserver agreement of PISA and VC for differentiating severe from non-severe MR. Eighteen cardiologists from 11 academic institutions interpreted PISA and VC from 16 patients [54]. A “kappa coefficient” of 1.0 indicates full agreement. Overall, interobserver agreement on the classification of MR as severe or non-severe was only fair for each of the study parameters. Raw agreement and kappa coefficient were 75 ± 16% and 0.32, respectively, for jet area, 75 ± 15% and 0.28 for VC measurements, and 78 ± 15% and 0.37 for PISA measurements. Hagendorff has also described the uncertainty of MR severity assessed by echocardiology [57, 58].

Doppler quantification of mitral regurgitation can be graded by the effective regurgitant orifice (ERO) area and volume overload, measured as regurgitant volume (RVol) [34, 59, 60]. These calculations can be difficult and affected by the multiple dynamic factors that influence MR severity: The 3D nature of the jet, the volume status, the left ventricular systolic pressure [the driving force], and the left atrial compliance [60, 61]. Machine settings such as Doppler gain and transducer frequency can also impact jet area [52]. However, quantitative grading of mitral regurgitation is a powerful predictor of the clinical outcome of asymptomatic mitral regurgitation [26]. Mild, moderate, and severe mitral regurgitation correspond to an effective regurgitant orifice area of less than 20 mm^2^, 20 to 39 mm^2^, and 40 mm^2^ or more, respectively. Patients with an ERO of at least 40 mm^2^ have severe MR and should be considered for surgery without delay [26]. This classification’s prognostic power supersedes that of all other semiquantitative indices.

## 5 LEFT VENTRICULAR RESPONSE TO MITRAL REGURGITATION AND SURGERY

The LV response to acute MR includes an increase in preload, a decrease in afterload, an increased ejection fraction (EF) and an increased total stroke volume [14]. These early compensatory changes are beneficial in the short term, yet persistent volume overload and neurohumoral stressor exposure drive maladaptive remodeling that transitions to decompensation, LV enlargement and dysfunction [7]. The progression from a compensated to a decompensated stage remains a poorly understood aspect of the pathophysiology of chronic primary MR. Gene expression changes during this transition have recently been described as an overexpression of pro-fibrotic genes [62]. Considering remodeling as a binary response may be simplistic. It is more likely that remodeling occurs across a continuum [13]. The transition may be the consequence of an increased regurgitant volume, a decrease in LV contractile function, an increase in afterload or some combination of these factors that, if not identified and corrected, progress to irreversible maladaptive remodeling [14].

The LV response to corrective surgery depends largely on its functional state before surgery and the surgical procedure [1,14,63]. LV volumes and systolic function determined by LVEF are late markers of myocardial dysfunction. Early MV repair of asymptomatic severe primary MR may prevent progression from a normal ventricle to a dysfunctional one. However, some patients with asymptomatic primary MR and preserved LVEF already exhibit changes in LV shape, impaired cardiac reserve, and limited cardio-pulmonary performance [36-38]. Ideally, in asymptomatic patients with severe primary MR, surgery should occur when the LVEF is normal but myocardial dysfunction is imminent. Identifying the first subtle, reversible, subclinical change in the left ventricle is fundamental for deciding the proper timing of early surgical intervention to ensure normal postoperative ventricular performance.

The right ventricular [RV]. response to primary mitral regurgitation was studied in patients referred for valve surgery when surgical timing was determined by symptoms and LV dysfunction [64]. Preoperative RV functional impairment was frequent (30%) during that era. The main determinant of RV dysfunction in patients with primary mitral regurgitation was left ventricular remodeling; LV enlargement and abnormal LV septal function thought to be related to ventricular interdependence [64, 65]. A 2020 study evaluated changes in RV function during exercise (bicycle ergometry) stress in patients with asymptomatic primary moderate to severe MR and preserved LVEF and compared them to a normal control group. Speckled tracking echocardiography measured RV and LV longitudinal strain and strain rate at rest, during stress and recovery in both groups. Conventional parameters of RV function and RV myocardial deformation did not significantly differ between the groups at all stages of stress (*p*>0.05), and systolic pulmonary artery pressure did not correlate with RV function. In these patients with primary mitral regurgitation and preserved LV function, the most important determinant of RV function during stress was LV function [66].

## 6 LEFT VENTRICULAR SHAPE

The left ventricular shape is the first metric to change during early LV compensation for primary MR and occurs before irreversible LV dysfunction develops [5, 6, 67]. LV shape is fundamentally related to LV performance. In normal subjects, each ventricular contraction is associated with a progressive reduction in LV volume and a change to a more elliptical systolic shape. This cardiac cycle shape change at rest is augmented during exercise. LV shape is an independent determinant of exercise capacity [68]. LV systolic and diastolic shape at peak exercise and the change in shape during exercise strongly correlate with exercise endurance. Patients with more spherical ventricles have a significant reduction in exercise capacity [68]. Tomlinson, in 1987, showed that the reduced ability of the heart to develop an elliptical shape during systole is an early indicator of impaired systolic function [69].

The spherical LV shape associated with secondary functional MR occurs after global LV remodeling, eccentric hypertrophy, enlargement, and dysfunction have developed. The abnormal mitral leaflet coaptation and functional MR are caused by the LV spherical shape and are not the result of simple LV chamber enlargement [70, 71]. The early, subtle, diastolic LV sphericity that occurs to compensate for primary MR, described below, develops before LV dysfunction as an adaptive response to MR that increases the stroke volume and maintains hemodynamic stability. The mechanism of these secondary and primary spherical shape changes is significantly different.

In patients with compensated primary MR and normal LVEF, the left ventricle maintains normal forward flow by increasing the stroke volume. The larger stroke volume is a consequence of increased diastolic filling; the characteristic of the systolic ejection resembles that of normal subjects. However, diastole is significantly different [5]. Gibson, in 1975, studied continuous changes in LV shape during the cardiac cycle in normal and abnormal ventricles, and described an early diastolic spherical LV shape resulting from an asymmetric expansion in the short axis dimension, causing a rapid non-uniform diastolic filling, in subjects with severe primary MR. This change precedes the long-axis dimension change, which is due to the upward movement of the aortic root and the outward movement of the apex [5]. Ross, in 1974, used a frame-by-frame analysis of left ventriculograms to continuously assess the LV shape during the cardiac cycle in 10 normal subjects and 40 patients with heart disease and reported that the LV adaptation to a chronic volume overload is mediated through normal performance of an enlarged cardiac circumference [6].

**Fig. (1):**
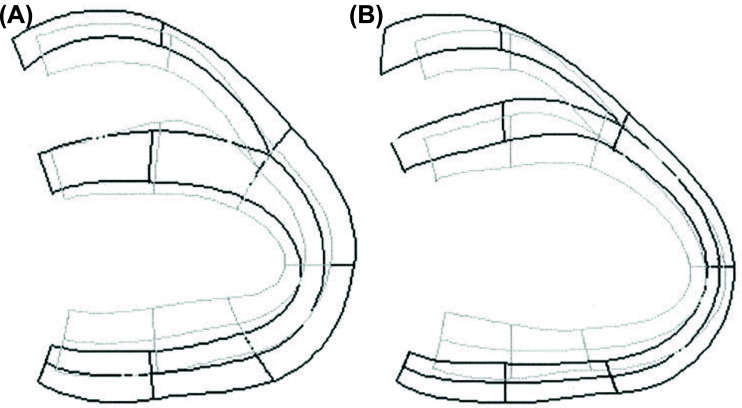
Cardiac shape in normal and MR contractions. Light lines are systole, and dark lines are diastole. (**A**) is normal. (**B**) shows change with MR: Note the diastolic shift of septum into RV, increasing LV end-diastolic volume.-In systole, the augmented septal contraction into the LV increases stroke volume and maintains cardiac output, despite the regurgitant volume loss. **Source:** Reprinted with permission from Am J Physiol 1996;271:H2689-700.Young AA, Orr R, Smaill BH, Dell'Italia LJ. Three-dimensional changes in left and right ventricular geometry in chronic mitral regurgitation with permission from The American Physiological Society.

Young, in 1996, examined changes in left and right (RV). ventricular geometry during chronic MR [68]. Ventricular shape changes were quantified with a 3D finite-element model fitted to chamber contours traced on cardiac magnetic resonance images. Eight dogs were studied before and 5-6 months after induction of MR caused by percutaneous chordal rupture of the mitral apparatus (Fig. **1**). MR increased LV end-diastolic volume (LVEDV; 99 *vs.* 57 ml; *p*<0.001) and LV stroke volume (LVSV; 55 *vs.* 25 ml; *p*<0.001) in contrast, RV end-diastolic volume decreased (RVEDV; 45 *vs.* 55 ml; *p*<0.01), whereas RV stroke volume was maintained. Shape changes due to MR were characterized by a marked (7.4 mm) rightward shift of the septum relative to the lateral LV free wall at end-diastole. In contrast, the distance from the RV-free wall to the lateral LV-free wall was relatively unchanged. During systole, the displacement of the septum into the LV increased significantly (7.3 *vs.* 2.9 mm; *p*<0.01), consistent with the end-diastolic dimension change [67]. Thus, chronic severe (chordal rupture). primary MR produces an asymmetric LV dilation of the short axis dimension during diastole, with preferential expansion in the lateral-septal direction, resulting in a more spherical LV diastolic shape. This is accomplished by a substantial diastolic shift of the interventricular septum into the RV cavity. In systole, the septum moves significantly leftward, contributing to the increased LV stroke volume. The RV stroke volume is maintained despite the decreased RVEDV.

An elegant 2010 surgical study assessed serial changes in left ventricular performance and 3-dimensional (3D). shape in 50 patients with severe asymptomatic primary mitral valve regurgitation and normal ejection fractions (>55%), before and after mitral valve repair [38]. Transthoracic 3D echocardiography was performed the day before and 6 and 12 months after mitral valve repair. An age-matched control group of 50 normal subjects was studied for comparison. There were modifications in the end-diastolic LV shape: Before surgery, compared to normal controls, sphericity was augmented and conicity was decreased; at 6 months, these shape changes were reversed. This study confirmed [1]. patients with asymptomatic severe primary mitral valve regurgitation and preserved LV function already exhibit changes in LV shape, [2] there is favorable remodeling in the LV shape from an abnormal spherical to a more normal conical shape at 6 months following early mitral valve repair.

**Fig. (2):**
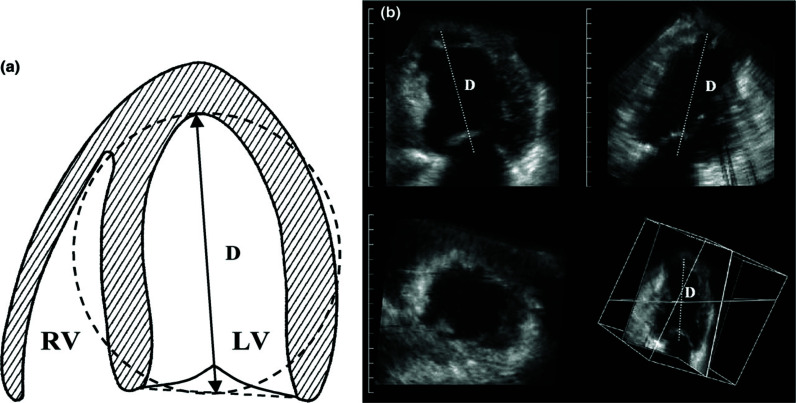
(**a**). Schematic drawing of calculation of the 3D sphericity index. The LV cavity is shown, of which *D* is the LV end-diastolic major long axis. With the formula: [4/3∗π∗[D/2]^3^ a spherical volume in mL can be calculated, of which *D* is the diameter (cm). The 3D sphericity index is calculated as EDV/[4/3∗π∗[D/2]^3^. (**b**). Four-tile image display of the dynamic 3D dataset with two near perpendicular long axes (top panels), a short axis [lower left], and a cubical display with the corresponding cut planes (lower right). The measurement of *D* is shown. A prominent trabecula is present in the LV apex. Reprinted from Eur Heart J 2004;25:680-7). Mannaerts HF, van der Heide JA, Kamp O, Stoel MG, Twisk J, Visser CA. Early identification of left ventricular remodelling after myocardial infarction, assessed by transthoracic 3D echocardiography with permission from Oxford University Press.

LV shape is most accurately described by measuring the left ventricular sphericity index (LV-Si) from 3D LV volumes rather than 2D cross-sectional areas [72, 73]. The 3D derived LV-Si is calculated by dividing the LV end-diastolic volume (calculated from a 3D dataset) by the volume of a sphere, the diameter of which is equal to the LV end-diastolic long axis dimension (Fig. **2**) [74, 75]. A large comprehensive 3D echocardiographic analysis of LV geometry in healthy adult subjects established age-specific and gender-specific reference ranges and cut off values for LV volumes, mass, and sphericity [71]. However, values for normal LV geometry may not apply to patients with severe primary MR. A small, but reversible change in LV sphericity is a sign of ventricular remodeling, therefore, early evidence of subclinical decompensation. Among patients with severe asymptomatic primary MR and normal LV function, brain natriuretic peptide [BNP]. levels at baseline independently identified patients who are at high risk. [76, 77]. BNP is a hormone secreted primarily by the ventricular myocardium in response to cardiac stress [78, 79]. BNP levels are simple and objective measures of cardiac function. In heart failure patients, symptomatic or asymptomatic, each 100 pg/ml increase in BNP was associated with a 35% increase in the relative risk of death [79]. Serial measurements of BNP have been shown to predict outcome in patients with severe asymptomatic primary MR independently [80]. Perhaps an additional biochemical marker (BNP) should be combined with the sphericity index for a more comprehensive serial assessment of the evolving left ventricular response to volume overload.

Software is available for 3D calculation of the LV sphericity index [81]. LV-Si has been shown to be an accurate and an early (acute phase) predictor of remodeling in patients following acute myocardial infarction [75]. LV-Si has also be utilized to differentiate between an acute phase Takotsubo syndrome (TS) and an acute phase anterior myocardial infarction, TS having a higher LV-Si indicating a more spherical left ventricle [82].

## 7 DISCUSSION

Primary mitral regurgitation is a consequence of mitral leaflet myxomatous degeneration. MR results in left ventricular volume overload, as blood is ejected both forward into the aorta and backward into the left atrium. Patients with severe primary MR may remain asymptomatic for years because of compensatory changes in left ventricular shape, myocardial contractility and left atrial compliance. However, persistent volume overload or progression, or if physiologic adaptive mechanisms fail, then global LV chamber enlargement and increased wall stress result in maladaptive, irreversible eccentric remodeling.

The contemporary recommended therapy for patients with asymptomatic severe primary MR and normal LV function is early mitral valve repair in centers of excellence by proficient surgeons. Older strategies for the timing of surgical intervention, such as the onset of symptoms or cardiac enlargement, are associated with excess postoperative morbidity and mortality, primarily from LV dysfunction and heart failure. Once symptoms of LV dysfunction develop, MR has already transitioned to a decompensated stage. Early mitral valve repair, during the compensated stage of MR, preserves LV function, and is associated with low operative mortality, greater long-term survival, and an improved quality of life.

Early research on primary mitral valve regurgitation, cited above, discovered that the first left ventricular remodeling response to mitral regurgitation is a regional, diastolic short-axis dilation that increases diastolic volume and creates a more spherical shape [5, 6, 67]. This increase in LV diastolic volume augments the stroke volume during systolic ejection. The larger stroke volume is an adaptive response to maintain normal forward flow in the face of the lost regurgitant volume. The increased LV diastolic volume develops from an asymmetric short-axis expansion caused by a marked rightward diastolic shift of the interventricular septum into the right ventricular cavity. During systole, an augmented septal contraction moves significantly leftward, into the left ventricular cavity, contributing to the larger stroke volume. These adaptive geometric changes during the cardiac cycle, the creation of a more spherical ventricular shape, is the heart's initial mechanism to stabilize hemodynamics.

A 2010 surgical study of mitral valve repair in patients with asymptomatic severe primary mitral regurgitation and normal left ventricular ejection fraction (LVEF >55%), confirmed that these asymptomatic patients already exhibited early ventricular remodeling with an abnormal end-diastolic LV shape. Before surgery, the patients LV sphericity was augmented and conicity was decreased, as compared to normal controls [38]. Surgery modified the end-diastolic LV shape. At 6 months after surgery, these changes were reversed. Early MV repair led to the return of a near-normal conical LV shape and confirmed the muscle viability of the previously spherical ventricle. The authors stated that the advantage of LV shape analysis lies in its capacity to quantitatively describe subtle changes in LV morphology due to LV remodeling, earlier than conventional LV volume analysis.

## CONCLUSION

Primary mitral regurgitation is the most common heart valve disease in the USA. These patients often remain asymptomatic for many years while the chronic volume overload progressively transforms the normal left ventricle from a compensated to a decompensated, enlarged ventricle with eccentric hypertrophy and irreversible global LV dysfunction.

During the early asymptomatic compensated stage of primary MR, there is reversible remodeling of the left ventricular shape. The left-ventricle adjusts to the loss of the regurgitant volume by increasing the stroke volume which maintains normal forward flow and hemodynamic stability. This is accomplished by an asymmetric increase in the short-axis diastolic dimension, that develops from a marked diastolic rightward shift of the interventricular septum into the right ventricular cavity, thus enlarging the LV diastolic volume and creating a more spherical shape. During systole, the augmented leftward displacement of the septum into the left ventricle generates a larger stroke volume. The early adaptive, reversible, spherical shape change is the remodeling response that increases stroke volume, maintains a normal cardiac output and preserves the patient’s asymptomatic status. It can be measured, quantified, and serially followed by the LV-Si and may potentially be used to define the optimal surgical timing for asymptomatic patients.

## LIST OF ABBREVIATIONS

EF: Ejection Fraction
ERO: Regurgitant Orifice Area
MR: Mitral Regurgitation
PISA: Proximal Isovelocity Surface Area
VC: Vena Contracta

## References

[1] Enriquez-Sarano M., Akins C.W., Vahanian A. (2009). Mitral regurgitation.. Lancet.

[2] Apostolidou E., Maslow A.D., Poppas A. (2017). Primary mitral valve regurgitation: Update and review.. Glob. Cardiol. Sci. Pract..

[3] Enriquez-Sarano M., Schaff H.V., Orszulak T.A., Tajik A.J., Bailey K.R., Frye R.L. (1995). Valve repair improves the outcome of surgery for mitral regurgitation. A multivariate analysis.. Circulation.

[4] Mick S.L., Keshavamurthy S., Gillinov A.M. (2015). Mitral valve repair versus replacement.. Ann. Cardiothorac. Surg..

[5] Gibson D.G., Brown D.J. (1975). Continuous assessment of left ventricular shape in man.. Heart.

[6] Ross J (1974). Adaptations of the left ventricle to chronic volume overload.. Circ Res.

[7] Hill J.A., Olson E.N. (2008). Cardiac plasticity.. N. Engl. J. Med..

[8] Opie L.H., Commerford P.J., Gersh B.J., Pfeffer M.A. (2006). Controversies in ventricular remodelling.. Lancet.

[9] Pitoulis F.G., Terracciano C.M. (2020). Heart plasticity in response to pressure- and volume-overload: A review of findings in compensated and decompensated phenotypes.. Front. Physiol..

[10] El Sabbagh A., Reddy Y.N.V., Nishimura R.A. (2018). Mitral valve regurgitation in the contemporary era.. JACC Cardiovasc. Imaging.

[11] Li F., Wang X., Capasso J.M., Gerdes A.M. (1996). Rapid transition of cardiac myocytes from hyperplasia to hypertrophy during postnatal development.. J. Mol. Cell. Cardiol..

[12] Porrello E.R., Widdop R.E., Delbridge L.M.D. (2008). Early origins of cardiac hypertrophy: does cardiomyocyte attrition programme for pathological ‘catch-up’ growth of the heart?. Clin. Exp. Pharmacol. Physiol..

[13] Dorn G.W. (2007). The fuzzy logic of physiological cardiac hypertrophy.. Hypertension.

[14] Gaasch W.H., Meyer T.E. (2008). Left ventricular response to mitral regurgitation: Implications for management.. Circulation.

[15] Grossman W., Jones D., McLaurin L.P. (1975). Wall stress and patterns of hypertrophy in the human left ventricle.. J. Clin. Invest..

[16] Grossman W., Paulus W.J. (2013). Myocardial stress and hypertrophy: A complex interface between biophysics and cardiac remodeling.. J. Clin. Invest..

[17] Lorell B.H., Carabello B.A. (2000). Left ventricular hypertrophy: Pathogenesis, detection, and prognosis.. Circulation.

[18] Carabello B.A. (2002). Concentric versus eccentric remodeling.. J. Card. Fail..

[19] Carabello B.A., Zile M.R., Tanaka R., Cooper G I.V. (1992). Left ventricular hypertrophy due to volume overload versus pressure overload.. Am. J. Physiol..

[20] Corin W.J., Monrad E.S., Murakami T., Nonogi H., Hess O.M., Krayenbuehl H.P. (1987). The relationship of afterload to ejection performance in chronic mitral regurgitation.. Circulation.

[21] de Simone G. (2004). Concentric or eccentric hypertrophy: How clinically relevant is the difference?. Hypertension.

[22] Gerdes A.M. (2002). Cardiac myocyte remodeling in hypertrophy and progression to failure.. J. Card. Fail..

[23] Griffin B.P. (2006). Timing of surgical intervention in chronic mitral regurgitation: Is vigilance enough?. Circulation.

[24] Ren J.F., Kotler M.N., Depace N.L. (1983). Two-Dimensional echocardiographic determination of left atrial emptying volume: A noninvasive index in quantifying the degree of nonrheumatic mitral regurgitation.. J. Am. Coll. Cardiol..

[25] Braunwald E., Awe W.C. (1963). The syndrome of severe mitral regurgitation with normal left atrial pressure.. Circulation.

[26] Enriquez-Sarano M., Avierinos J.F., Messika-Zeitoun D. (2005). Quantitative determinants of the outcome of asymptomatic mitral regurgitation.. N. Engl. J. Med..

[27] Rosenhek R., Rader F., Klaar U. (2006). Outcome of watchful waiting in asymptomatic severe mitral regurgitation.. Circulation.

[28] Selzer A., Katayama F. (1972). Mitral regurgitation: Clinical patterns, pathophysiology and natural history.. Medicine.

[29] Tribouilloy C.M., Enriquez-Sarano M., Schaff H.V. (1999). Impact of preoperative symptoms on survival after surgical correction of organic mitral regurgitation: Rationale for optimizing surgical indications.. Circulation.

[30] Enriquez-Sarano M. (2002). Timing of mitral valve surgery.. Br. Heart J..

[31] Delahaye J.P., Gare J.P., Viguier E., Delahaye F., De Gevigney G., Milon H. (1991). Natural history of severe mitral regurgitation.. Eur. Heart J..

[32] Rosen S.E., Borer J.S., Hochreiter C. (1994). Natural history of the asymptomatic/minimally symptomatic patient with severe mitral regurgitation secondary to mitral valve prolapse and normal right and left ventricular performance.. Am. J. Cardiol..

[33] Avierinos J.F., Gersh B.J., Melton L.J. (2002). Natural history of asymptomatic mitral valve prolapse in the community.. Circulation.

[34] Enriquez-Sarano M., Seward J.B., Bailey K.R., Tajik A.J. (1994). Effective regurgitant orifice area: A noninvasive Doppler development of an old hemodynamic concept.. J. Am. Coll. Cardiol..

[35] Ling L.H., Enriquez-Sarano M., Seward J.B. (1996). Clinical outcome of mitral regurgitation due to flail leaflet.. N. Engl. J. Med..

[36] Lee R., Haluska B., Leung D.Y., Case C., Mundy J., Marwick T.H. (2005). Functional and prognostic implications of left ventricular contractile reserve in patients with asymptomatic severe mitral regurgitation.. Heart.

[37] Messika-Zeitoun D., Johnson B.D., Nkomo V. (2006). Cardiopulmonary exercise testing determination of functional capacity in mitral regurgitation: Physiologic and outcome implications.. J. Am. Coll. Cardiol..

[38] Maffessanti F., Caiani E.G., Tamborini G. (2010). Serial changes in left ventricular shape following early mitral valve repair.. Am. J. Cardiol..

[39] Ott D.A. (1995). Repairing the mitral valve.. Circulation.

[40] Lazam S., Vanoverschelde J.L., Tribouilloy C. (2017). Twenty-year outcome after mitral repair versus replacement for severe degenerative mitral regurgitation.. Circulation.

[41] Kim H.M., Cho G.Y., Hwang I.C. (2018). Myocardial strain in prediction of outcomes after surgery for severe mitral regurgitation.. JACC Cardiovasc. Imaging.

[42] Pastore M.C., Mandoli G.E., Dokollari A. (2022). Speckle tracking echocardiography in primary mitral regurgitation: Should we reconsider the time for intervention?. Heart Fail. Rev..

[43] Witkowski T.G., Thomas J.D., Debonnaire P.J.M.R. (2013). Global longitudinal strain predicts left ventricular dysfunction after mitral valve repair.. Eur. Heart J. Cardiovasc. Imaging.

[44] Kislitsina O.N., Thomas J.D., Crawford E. (2020). Predictors of left ventricular dysfunction after surgery for degenerative mitral regurgitation.. Ann. Thorac. Surg..

[45] Bijvoet G.P., Teske A.J., Chamuleau S.A.J., Hart E.A., Jansen R., Schaap J. (2020). Global longitudinal strain to predict left ventricular dysfunction in asymptomatic patients with severe mitral valve regurgitation: Literature review.. Neth. Heart J..

[46] Canessa M., Thamman R., Americo C., Soca G., Dayan V. (2021). Global longitudinal strain predicts survival and left ventricular function after mitral valve surgery: A meta-analysis.. Semin. Thorac. Cardiovasc. Surg..

[47] Kim M.S., Kim Y.J., Kim H.K. (2009). Evaluation of left ventricular short- and long-axis function in severe mitral regurgitation using 2-dimensional strain echocardiography.. Am. Heart J..

[48] Maciver D.H. (2012). The relative impact of circumferential and longitudinal shortening on left ventricular ejection fraction and stroke volume.. Exp. Clin. Cardiol..

[49] Marwick T.H. (2006). Measurement of strain and strain rate by echocardiography: Ready for prime time?. J. Am. Coll. Cardiol..

[50] Otto C.M., Nishimura R.A., Bonow R.O. (2021). 2020 ACC/AHA guideline for the management of patients with valvular heart disease: A report of the American college of cardiology/American heart association joint committee on clinical practice guidelines.. Circulation.

[51] Grayburn P.A., Weissman N.J., Zamorano J.L. (2012). Quantitation of mitral regurgitation.. Circulation.

[52] Zoghbi W.A., Adams D., Bonow R.O. (2017). Recommendations for noninvasive evaluation of native valvular regurgitation.. J. Am. Soc. Echocardiogr..

[53] Apostolakis E.E., Baikoussis N.G. (2009). Methods of estimation of mitral valve regurgitation for the cardiac surgeon.. J. Cardiothorac. Surg..

[54] Biner S., Rafique A., Rafii F. (2010). Reproducibility of proximal isovelocity surface area, vena contracta, and regurgitant jet area for assessment of mitral regurgitation severity.. JACC Cardiovasc. Imaging.

[55] Buck T., Plicht B., Kahlert P., Schenk I.M., Hunold P., Erbel R. (2008). Effect of dynamic flow rate and orifice area on mitral regurgitant stroke volume quantification using the proximal isovelocity surface area method.. J. Am. Coll. Cardiol..

[56] Roberts B.J., Grayburn P.A. (2003). Color flow imaging of the vena contracta in mitral regurgitation: Technical considerations.. J. Am. Soc. Echocardiogr..

[57] Hagendorff A., Knebel F., Helfen A. (2021). Echocardiographic assessment of mitral regurgitation: Discussion of practical and methodologic aspects of severity quantification to improve diagnostic conclusiveness.. Clin. Res. Cardiol..

[58] Hagendorff A., Stöbe S. (2022). Plausible functional diagnostics by rational echocardiography in the assessment of valvular heart disease - Role of quantitative echocardiography in the assessment of mitral regurgitation.. Front. Cardiovasc. Med..

[59] Enriquez-Sarano M., Bailey K.R., Seward J.B., Tajik A.J., Krohn M.J., Mays J.M. (1993). Quantitative Doppler assessment of valvular regurgitation.. Circulation.

[60] Zoghbi W., Enriquez-Sarano M., Foster E. (2003). Recommendations for evaluation of the severity of native valvular regurgitation with two-dimensional and doppler echocardiography.. J. Am. Soc. Echocardiogr..

[61] Zhou X., Vannan M.A., Lancellotti P. (2016). Quantitative three-dimensional color flow echocardiography of chronic mitral regurgitation: New methods, new perspectives, new challenges.. J. Am. Soc. Echocardiogr..

[62] Tsai F.C., Chen Y.L., Yen K.C. (2021). Gene expression changes of humans with primary mitral regurgitation and reduced left ventricular ejection fraction.. Int. J. Mol. Sci..

[63] Bonow R.O., Adams D.H. (2016). The time has come to define centers of excellence in mitral valve repair.. J. Am. Coll. Cardiol..

[64] Le Tourneau T., Deswarte G., Lamblin N. (2013). Right ventricular systolic function in organic mitral regurgitation: Impact of biventricular impairment.. Circulation.

[65] Weber K.T., Janicki J.S., Shroff S., Fishman A.P. (1981). Contractile mechanics and interaction of the right and left ventricles.. Am. J. Cardiol..

[66] Žvirblytė R, Merkytė I, Tamulėnaitė E (2020). Right ventricle mechanics and function during stress in patients with asymptomatic primary moderate to severe mitral regurgitation and preserved left ventricular ejection fraction.. Medicina.

[67] Young A.A., Orr R., Smaill B.H., Dell’Italia L.J. (1996). Three-dimensional changes in left and right ventricular geometry in chronic mitral regurgitation.. Am. J. Physiol..

[68] Tischler M.D., Niggel J., Borowski D.T., Lewinter M.M. (1993). Relation between left ventricular shape and exercise capacity in patients with left ventricular dysfunction.. J. Am. Coll. Cardiol..

[69] Tomlinson C.W. (1987). Left ventricular geometry and function in experimental heart failure.. Can. J. Cardiol..

[70] Kono T., Sabbah H.N., Rosman H., Alam M., Jafri S., Goldstein S. (1992). Left ventricular shape is the primary determinant of functional mitral regurgitation in heart failure.. J. Am. Coll. Cardiol..

[71] Sabbah H.N., Kono T., Rosman H., Jafri S., Stein P.D., Goldstein S. (1992). Left ventricular shape: A factor in the etiology of functional mitral regurgitation in heart failure.. Am. Heart J..

[72] Monaghan M.J. (2006). Role of real time 3D echocardiography in evaluating the left ventricle.. Heart.

[73] Schiros C.G., Dell’Italia L.J., Gladden J.D. (2012). Magnetic resonance imaging with 3-dimensional analysis of left ventricular remodeling in isolated mitral regurgitation: Implications beyond dimensions.. Circulation.

[74] Ambale-Venkatesh B., Yoneyama K., Sharma R.K. (2017). Left ventricular shape predicts different types of cardiovascular events in the general population.. Heart.

[75] Mannaerts H., van der Heide J.A., Kamp O., Stoel M.G., Twisk J., Visser C.A. (2004). Early identification of left ventricular remodelling after myocardial infarction, assessed by transthoracic 3D echocardiography.. Eur. Heart J..

[76] McCullough P.A., Hanzel G.S. (2009). B-type natriuretic peptide and echocardiography in the surveillance of severe mitral regurgitation prior to valve surgery.. J. Am. Coll. Cardiol..

[77] Pizarro R., Bazzino O.O., Oberti P.F. (2009). Prospective validation of the prognostic usefulness of brain natriuretic peptide in asymptomatic patients with chronic severe mitral regurgitation.. J. Am. Coll. Cardiol..

[78] Doust J., Lehman R., Glasziou P. (2006). The role of BNP testing in heart failure.. Am. Fam. Physician.

[79] Doust J.A., Pietrzak E., Dobson A., Glasziou P. (2005). How well does B-type natriuretic peptide predict death and cardiac events in patients with heart failure: Systematic review.. BMJ.

[80] Klaar U., Gabriel H., Bergler-Klein J. (2011). Prognostic value of serial B‐type natriuretic peptide measurement in asymptomatic organic mitral regurgitation.. Eur. J. Heart Fail..

[81] Muraru D., Badano L.P., Peluso D. (2013). Comprehensive analysis of left ventricular geometry and function by three-dimensional echocardiography in healthy adults.. J. Am. Soc. Echocardiogr..

[82] Khanna S., Bhat A., Chen H.H., Tan J.W.A., Gan G.C.H., Tan T.C. (2020). Left ventricular sphericity index is a reproducible bedside echocardiographic measure of geometric change between acute phase Takotsubo’s syndrome and acute anterior myocardial infarction.. Int. J. Cardiol. Heart Vasc..

